# Interpretation of Opposite-Sex Friendship Based on Social Ecology Model in Iranian Females

**Published:** 2012

**Authors:** Seyed Abbas Mousavi, Afsaneh Keramat, Katayon Vakilian, Reza Chaman

**Affiliations:** 1 Department of Psychiatry, Shahroud Medical Science University, Shahroud, Iran.

**Keywords:** Female Students, Friendship, Iran, Opposite-Sex, Social Ecology

## Abstract

**Objective:** One of the main cultural issues in universities is relationships between male and female students. Due to the adverse consequences of this issue, explaining of the beyond the causes of friendship with opposite-sex, is one of the first steps in planning for youth issues.

**Methods:** This qualitative research was conducted based on content analysis. Nineteen female university students were enrolled. Semi-structured questions through four sessions were used to gather required data. Two themes were extracted. One was the behaviors leading friendship which consisted of 5 subsets including self and extra-self, meso, exo-, and macro-systems. The other theme was the actions against with consequences of sex which are made of 2 subsets of the quality and the consequences of sex.

**Results:** Various factors such as person attitude, felling lonely, and community atmosphere, including the university environment, family, friends, religious beliefs and media that promote the Western culture can be effective in friendship before marriage.

**Conclusion: **Since the society of our country is considered a young populated one and the culture of the West through the media currently influences on our culture, reproductive health programs should be developed in a high priority focusing on youth fitted with their current needs according to Islamic-Iranian culture.

## Introduction

University students are considered an important part of each society and paying attention to their healthy behaviors including reproductive health behaviors should be taken into consideration. 

According to statistics issued by the Centers for Disease Control and Prevention (CDC), prevalence of health-threatening behaviors including smoking, alcohol drinking, high-risk sexual behaviors, and drug abuse is growing in youth population (1). A study in Tehran among high school students showed that 2.2% had sexual contacts (2). Another study in Iran showed that 32% of those with sexual intercourse experience had more than two partners and 39.8% of them did not use condom (3).

One of the major health concerns which can affect young people is human immunodeficiency virus (HIV) infection. 

 The total number of registered HIV/AIDS infected cases in Iran was about 7,510 persons by late September 2004, and this figure reached to 23,125 in 2011 (4).

In recent years, due to expanding urbanization and pervasive mass media, new patterns of communication between male and female youngsters have emerged (5-7). University students’ behavior is considered as one of the important patterns of behaviors in each society. Understanding their needs, values and norms can help researchers to anticipate some of the social realities (6). 

Hence, this study with its nature-oriented perspective intended to explain the pattern of opposite-sex friendship in a group of Iranian female students. We used focus groups discussion (FGD) because it is a form of group interview that provides communication between research participants in order to provide information required (8). FGD is a form of group interview that provide communication between research participants in order to generate data (9). It is useful for exploring people's knowledge and attitude (10). FGD is particularly appropriate when the interviewer has a series of open-ended questions and wants to encourage participants to explore the issues of importance to them generating their own questions and pursuing their own priorities (11). Social ecological model (SEM) was used to analyze data. It can provide a theoretical framework to analyze various contexts in multiple types of research and in communications (12). SEM is the study of people in an environment and the influences on one another (13). It is primarily a qualitative research model to conduct field observations; however, it has been and can also be utilized in experimental settings. In SEM, the effective factors on behavior occur in micro-, meso-, exo- and macro-systems, in which individual, interpersonal, organizational factors, community and culture are explained (14).

## Materials and Methods

Qualitative research projects are based on a nature-oriented paradigm that focuses on the principles of reality (15).

Data in this study were analyzed based on content analysis (CA). CA is a systematic method of classification and coding that can be used in text information discovery, and in revealing patterns of words used, their frequency and structure, relationship between them, and communication analysis (16).  

CA, beyond extracting data from text content, can be used to reveal the themes and patterns Therefore, in this study, using this method and based on the descriptions given by the participants, overt and hidden concepts specified by coded, summarized, and classified concepts and themes were extracted. Codes were derived based on meaningful units of the descriptions of participants, and then classified based on differences or similarities (17).  

Nineteen students participated in this study. FGD was conducted in four sessions with a maximum of 90 minute. Before entering the interview, they were informed of the content of discussions.  

Interview began with two general questions. Do you have friends who had experience of friendship with opposite sex? In your opinion, how is the trend of relationships between male and female students in universities in Iran? After that, according to the interview process, probe questions were asked.  

Interviews were recorded and personal characteristics of the subjects were registered. When the new data as a new code and a new category were not extracted, it indicated that the data is saturated, then no more meetings were scheduled (18, 19). Verbal communications of the participants, which were recorded on tape, together with non-verbal communications were transcribed.  Interviews were reviewed several times, then, the text pieces were broken into units of meaning. Then summarized semantic units and codes were extracted in the form of words or phrases. Codes, based on the conceptual and semantic similarity, were classified. Decreasing trend of data in all stages including sub-categories and main categories was moving until not only conceptual units were summarized, but also the main categories and sub-categories became more abstract and conceptual. Credibility was established mainly through member checking. Member checking was used in 3 ways at 3 stages of data collection and data analysis: (1) at the pilot stage, the interviewer discussed the interview questions with participants at the end of each interview; (2) during formal interviews, the interviewer fed ideas back to participants to refine, rephrase, and interpret; (3) in an informal post-interview session, each participant was given the chance to discuss the findings (20). The dependability of the research was done by the supervisors review, the text of some interviews, codes and categories derived, were analyzed by several faculty members, which there was 92% agreement in the extracted results. In order to increase transferability of statuses to other groups, different participants were used in terms of their socioeconomic, educational level, and rich description about reporting of the research process in material and method section (20). Ethical considerations such as obtaining informed consent during research, collecting methods and data recorder were respected.

## Results

Mean (standard deviation) age of the sample was 20 ±)2.1( years and none of them were working. 10%(2) were married, 80%(15) of them lived in small towns and the remaining 20%(4) lived in large cities. 70% (13) were residing in dormitories and others live with their parents. In analyzing the results, the researcher decided, regarding the classification of codes, to use the theory of social ecology. Two themes, as systems leading sexual behavior, and behavior coping with the consequences of sexual behavior were extracted.

*Sexual Behavior* *Leading* *Systems:*

Systems leading friendship was extracted from the four main classifications, namely self micro-system (1-1), micro-system extra self (1-2), university organization meso-system (1-3), the mass media exo-system (1-4) and values and religious beliefs macro-system (1-5) were extracted.

*1-1- Self Micro-systems:*

In this group, individual values and attitudes toward friendship problems were classified. This study showed that participants believed that relations before marriage are increasing and are considered a common issue. A participant stated, “At the university, the relationship before marriage between male and female students has become more prevalent than before. Boys and girls are given more freedom”. Another participant continued, "If there are no relationships between boys and girls, this is unusual”. 

In their opinion, students who do not have opposite-sex friendship will be rejected from their friends’ community. So, not being in isolation, there should be established relationships. It appears that students are trying to behave in mutual interactions in social environments; that is, university, based on their understanding of stereotypes and expectations of society. 

*1-2 Microsystems Extra Self:*

It was the second category of the main theme which included sub-classifications of family, emotional and sexual needs, same-sex friends’ atmosphere behavior of the opposite sex, and experience of previous friendship failures.

*1-2-1 Micro-system of family*. 

Expressing the cause of friendship, the majority of students take the family involved in this issue for various reasons. One of the reasons expressed by the majority of the students is that they believed various degrees of freedom given to female students by their families have a significant impact on this increasing process of communication, so that, one participant expressed, "Girls are allowed to travel to far places to study, they can have their own cell phones, and be very open-handed "; This means that young females are given much more freedom than before by their families. The second reason concerning the family involvement is that most families prohibit the youth from marriage at young age. The youth believe that marriage at young age may be appropriate; Why? Because there is evidence that Iran, along with decreasing age Menarche, age at first marriage has increased dramatically (21), and this situation along with other reasons that describes in this article may urge them to form pre-marriage friendships. One participant said, “The families’ expectations from their children have changed. First, they expect you to study, then find a good job and when getting a job, they tell you that life have become hard going”. Others expressed that sexual behavior is reinforced because of new family patterns, so that non-traditional families can be influential in this issue. 

On the other hand, male and female relationships in the family are taboo, especially adolescents do not usually talk with parents about these relationships, and the mothers are told that this is a closed form of relationship and they intend to get married; for example, we enter the relationship in an implicit form. While the reality may be something else, most relationships are formed out of sight of parents. One of the participants explains about his/her father, “Father is really scary”. The other said, "If mothers are told about our relation because we are going to get married, some mothers treat us well, and they help us to form our relationships correctly. But at the same time, fathers cannot be told about it any way”. While mothers are not fully aware of the relationship and its quality from one hand, the young are allowed to enter sexual relationships out of sight of the family from the other hand.

*1-2-2 Emotional Needs;*

Among other causes of making a relationship before marriage is emotional needs expressed by participants. They believe that because of being away from their families and parents’ job, their emotional needs are unmet, and they need to unbosom their thoughts with someone of the opposite sex because s/he is the right person to understand their feelings. One participant said "Most of times, it is a condition when someone is feeling lonely, then s/he wishes to talk with someone, (s)he often feels that if his/her friend is from the opposite sex, (s)he can reveal one’s thoughts and feelings to him/her. It seems that families do not allocate enough time to meet the emotional needs of young people. They need to be embraced by their family as well as their parents should show them enough affection.

*1-2-3 The Same-sex Friends Environment*: Friends and the norms governing on the group of friends are other important components in performing a behavior in people, and friendship, is not an exception from this rule. In this study, the participants considered role of friends effective in the performance of sexual behavior.

A participant told us about her experience, "We were with my sister on a trip and a young male gave me his phone number and I took it”. When asked the reason, “All my friends have boyfriends” she replied. Another participant said, ”Once you see two of your roommates have got married, or two of them have boyfriends, you’re observing them and as a sequence of keeping up with them, you yourself wish to have a boyfriend, too. So you take action to go toward it and you get the following".

*1-2-4 Behavior of the Opposite Sex:*

Female students generally believe that they might be blamed by their peers for not having friendship with males so that they get chances to achieve their sexual goals. One participant told ”One boy asks me to shake hands with me once you enter the classroom in front of our friends, so that I won’t be embarrassed in front of them” and another participant expressed that “If we do not behave based on what the boys wish, they will leave us”. It seems that girls are behaving under the pressure and urge of sexual behavior caused by their boyfriends. Lack of sufficient skills in ability to control a female against her sexual desires is one of the weak points among females that should be emphatically considered in reproductive health programs. 

*1-2-5- Broken Previous Experience:*

It seems that being badly hurt from the first relationship strengths the next relationships which are made only to intend to enjoy, delight and fun, and to forget the bitter memories of the past relationship. In these circumstances, girls makes friendship with other boys and even with several individuals at the same time and experience short-term friendships all of which are the consequences of failure in previous contacts. 

Such communication can put the person at the exposure of higher risk form psychological, social and biological aspects. One participant said, "The best relationship communication is always the first. Boys and girls try to think of marriage, but after failing, they just think of getting a date with their counterparts”.

*1-3:Meso system Organization of University:*

It seems values which govern the student community influence individual behavior and attitudes of students at the university level. In addition, individuals with their own behavior and attitudes, willingly or unwillingly, impose their values on the organization. In other words, it appears that university is an organization whose members individually help the aspects of relations outside of marriage, thus their expectations are provided as a meso-system. A participant said, "If you mean the society by the university, such relationships are not considered bad, it’s something natural that is needed. But, in the family or outside of university this behavior is not accepted”. 

*1-4: Exo-sytem of Mass Media;*

Another factor on which students had great emphasis is accessing to mass media such as private satellite, internet, pornographic pictures and movies showing or describing friendship and sexual behavior before marriage. Although, the media can inform young people about sexual and healthy sexual transmitting at the same time they can impose the values of Western societies, nations, and sexual ethics on the people of other countries threatening them. One of the participants said "The Iranian film series promote relations between boys and girls”.

*1-5: Macrosystem of values and laws governing community:*

Since our country is an Islamic country and the governing laws, common laws, as well as people’s values and beliefs are religious, religion can be effective on people's behavior. Participants believed that religion and its laws can be effective on sexual contacts between male and female students.

One participant said, "The role of religion is much stronger than friends”. Another one said “Some like to make friendship but not at the price of anything those who have loose and shaky faith may do so”. But unfortunately, it seems that Western culture with its own equipment like satellite and Mass Media intends to influence on our values and beliefs

*2: The actions against with consequences of sex:*

This theme is composed of two categories; the consequences of sex and sexual quality.

*2-1: The consequences of sex:*

In this study the consequences of guilt due to psychological trauma caused by the individual performance against the values and custom and norms of society were extracted.  Female sexual function develops a bad feeling of sin in them, which may be present for long periods of time. A participant expressed "Feeling guilty always accompanies human”. Also, being pregnant and consequences such actions like abortion, having teen hymen deflowered always cause physical or mental injuries in teenagers. One participant said "I saw a girl look for having an abortion”.

Marriage is usually a major problem for girls at the beginning of which the sexual behaviors start.

 . They perform actions such as the hymen repair surgery resulting in mainly high costs for them. 

The interviews revealed that most girls are afraid of pregnancy in comparison with sexually transmitted diseases (STD) like HIV infection, and being affected by the diseases are the last concerning, and such problems may increase the transfer of STD.

*2-2: Quality of Relationships:*

 Participants said that quality of relationships, depending on duration of their friendship, is different and its range varies from minimal physical relations to sexual intercourse. In other words, their friendship continued into stages that can be followed by physical or mental injury. But what our community has been more perilous is the kind of anal intercourse because the value of virginity is important among young people. One participant said "There is not the risk of pregnancy and on the other hand, the hymen remains intact". Meanwhile, sexual use in various ways for the girls and boys against its consequences is common, although there are ways to prevent of pregnancy (contraception) such as birth control pills and condoms, which they said are used by them who are concerned about pregnancy. Anal contact is a common approach without the problem of pregnancy and deflowering.

## Discussion

Microsystems including personal or interpersonal characteristics and group aspects of people make up their social identity Many of these features such as gender and ethnicity deeply ingrained in man and some others are learned through interacting with other individuals and groups. Individuals are continuously shaped not only by interacting with the environment and encountering various positions and problems, but also by coming in contact with other individuals (22). 

This study showed that students’ attitude toward the issue of male-female friendship before marriage is considered as a common practice in the university. Why? Because this relationship is common among society members in addition to this fact that if one does not make such a relationship, he will be rejected by his/her friends. One study showed that only 23.6 %of students have got a negative attitude toward relations before marriage (23). The issue of changing attitudes and values of Iranians, especially among young people, has been expressed explicitly in most social reports (7).  

Emergence of this phenomenon in our society can gradually lead families to have loose marriages and to face serious challenges. Increasing number of singles and rising marriage age, divorce rates, and illegitimate children are all examples of the consequences of this phenomenon (24).  

A study conducted among young people showed that men's motivation for this communication before marriage is sexual desires, and for girls has is getting physical care and support (25).  

In this study, emotional desires and loneliness have been mentioned as motivations for such behavior by the girls, and friendship with boys is known the best option to solve the problems of emotional desires and loneliness. This research indicated that talking with friends was one way that more than ninety percent of teenagers have done to keep mental health and reduce their depression. Also, 85% of teenagers have mentioned friends as the most effective sources who had helped them during depression periods (26).

In micro-systems extra self that includes friends and family, values and behaviors can be influenced (22). Former studies have shown that high level of parent and teen’s communication and conversation with low level of risky sexual intercourse are adversely associated (27, 28).  

Other studies showed that parental control and supervision are other factors that predict adolescent’s perilous sexual behavior (29, 30). On the other hand, warm relationship and control factors of family were recognized as important factors in preventing risky behaviors in female teenagers (29, 30).  On the other hand, warm relationship and control factors of family were recognized as important factors in preventing risky behaviors in female teenagers (31).  

 This study showed that parent’s communication with their children about premarital relations was very poor, so that fathers had been completely removed in this issue and even mothers are not aware of the quantity and quality of those relationships.  

Following religious and social traditions is one of the basic paradigms for Iranian society**. **But teenagers now live in such a period of time that they may reject most of these traditions and sometimes their behavior is against the norms of society, and these conflicting opinions cause them to be away from family and community values. Inability of teenagers to replace old traditional beliefs with new ones leads them to follow Western culture (32).  

In a study in Taiwan it was found that the inability of community to be in accordance with the transitional generation, poor communication with parents have increased incidence of unwilling teenage pregnancy (33). In addition, hazardous and unsafe sexual behavior in peer groups will be intensified (34, 35).

Due to support from the friends’ group, teen have more tendency toward them and usually are more interested into the wrong friends (32).  

Tendency to have relation with the opposite sex among teenagers has increased. However, awareness of negative consequences and logical control by families has been noted for such relationships (35).

Meso-systems are the organizational or institutional factors that form the environment within which the interpersonal relations occur. These aspects can be rules and policies within a more formal organization (22). It appears that university is an organization where the individuals help in dealing with the aspects of relations outside of marriage, thus provides its own expectations as a meso-system. People have tried in mutual interactions with social environments to act based on an understanding of stereotypes and expectations of society (36). 

 Thus, increasing friendship at the university level, one wishes to comply to meet the community's expectations; that is, here the university’s.

Mass Media were placed in exo-system. It refers to the influence of norms, standards, and social networks at the community level (22). Although it affects people, no individual is involved actively (14). This research showed that participants considered the role of Mass Media including private satellite important in promoting sexual behavior before marriage. Satellites, with producing and broadcasting programs at a large scale, have created a great development. Lack of control on the satellite waves have caused a large number of overseas programs enter the community and affect the intellectual atmosphere of personal and social dimensions (37).

 The findings of religious values and beliefs are placed in macro-systems. They have cultural contexts, not solely geographic, physical ones (14). Changes in attitudes and values along with friendship among male and female adolescents before marriage are a sign of massive cultural change that is emerging in our society. Research in Iran showed that the authority of religious values in male and female relationships becomes loosened day by day and people act based on secular-rational values. This means that people act based on their interests and profits instead of religious values (23). Other studies showed that changes in values and attitudes in among Iranians positively account for male and female relationships before marriage (38, 39).

Religious beliefs are part of Iranian society values. Religious standards often have got genuine moral features, which is believed that act on them reduces risky behavior (32). In this study, participants also recognized the role of religious believes in preventing high-risk sexual behaviors. 

But why, despite the governing religious culture, the trend of relations before marriage is growing without control. One reason could be cognitive compatibility, where their daily observations with their minds are in conflict with the religious principles; they try to match values and attitudes with their subjective experiences. Therefore, their values and attitudes will also change (40).

 Regarding research outcomes, findings showed that the physical consequences such as unwanted pregnancies and consequent abortions, the hymen injury and diseases such as HIV infection are threatening the females. A previous study in reported that 27.7% of Iranian males have sexual relations. Unwanted pregnancy is another concern which young girls are suffered.  Mental and emotional damages include losing confidence and self-esteem, depression, anxiety, loneliness and intellectual rumination about the relationship are among other major concerns (41).  

Informal access to information and international media such as satellite and internet help them to be exposed to sexual risk behaviors and a number of evidence shows that they sometimes involve a risky fertile and sexual behavior (42). 

## Conclusion

Since our country is considered a young populated one and Western culture is currently influencing our culture through the media, appropriate reproductive health programs should be developed according to young people’s needs based on Islamic-Iranian culture.

These programs should be made based on comprehensive studies on the formation of components of friendship with emphasis on appropriate solutions that can be provided to control them.  

## Authors’ Contributions

SAM conceived and designed the evaluation and helped to draft the manuscript. AK, KV and RCh participated in revising the manuscript. They reviewed codes and categories and presented their idea in this article. All authors participated in data analysis, read and approved the final manuscript.

## Notes

**Declaration of interest:** None.
